# A new model for *in situ* nitrogen incorporation into 4H-SiC during epitaxy

**DOI:** 10.1038/srep43069

**Published:** 2017-02-17

**Authors:** Gabriel Ferro, Didier Chaussende

**Affiliations:** 1 Université de Lyon, Université Claude Bernard Lyon1, CNRS, Laboratoire des Multimatériaux et Interfaces (LMI, UMR 5615), 43 bd du 11 Novembre 1918, F-69622 Villeurbanne, France; 2 Université Grenoble Alpes, CNRS, LMGP, F-38000 Grenoble, France; 3 Université Grenoble Alpes, CNRS, Institut Néel, F-38000 Grenoble, France

## Abstract

Nitrogen doping of 4H-SiC during vapor phase epitaxy is still lacking of a general model explaining the apparently contradictory trends obtained by different teams. In this paper, the evolutions of nitrogen incorporation (on both polar Si and C faces) as a function of the main growth parameters (C/Si ratio, temperature, pressure and growth rate) are reviewed and explained using a model based on surface exchanges between the gas phase and the uppermost 4H-SiC atomic layers. In this model, N incorporation is driven mainly by the transient formation of C vacancies, due to H_2_ etching, at the surface or near the surface. It is shown that all the growth parameters are influencing the probability of C vacancies formation in a similar manner as they do for N incorporation. The surface exchange model proposes a new framework for explaining the experimental results even beyond the commonly accepted reactor type dependency.

A new era in power electronics is currently being opened up following the strong development of wide band gap semiconductor materials. Among them, silicon carbide (SiC) plays the main role as can be seen from the recent achievement, at an industrial scale, of high quality, large size single crystalline wafers and epilayers^1^,^2^ which has promoted the spreading of high performance SiC based devices and modules. Despite the apparent maturity of SiC technology, most of the building blocks are, on a pure scientific footing, far from being thoroughly described and understood. This is the case of doping which is needed to tune SiC conductivity (n or p-type, or even semi-insulating). The dopants must incorporate on lattice site in order to be electrically active, in other words they should replace some of the atoms of the matrix and create bonds with the neighboring atoms. For SiC, which belongs to the IV-IV semiconductor compounds, though the incorporation site of each dopant will not affect the doping type since all the atoms of the matrix have the same valence, it may have some secondary effects like change in dopant concentration or even in dopant ionization energy. This is the case with boron or aluminum impurities which were shown to have different ionization energies whether they incorporate on Si or C site^3^,^4^,^5^. Furthermore, polytypism renders the picture more complex since the impurities can incorporate either on cubic or hexagonal sites (noted respectively k and h in Fig. 1) in addition to the choice between Si or C atomic sites. This leads to some (less pronounced) changes in ionization energies of the dopants^6^,^7^.

*In-situ* doping during crystal growth is very commonly used for controlling the doping of both the substrate and the thin or thick active layer(s) of SiC electronic devices. Assuming that dopant diffusion can be neglected in SiC, *in-situ* doping mechanism is restricted to a “pure surface problem”. The most admitted rule for impurity incorporation into SiC is the so-called “site competition” which was settled more than 20 years ago by the pioneer work of Larkin *et al*.^8^. Basically, the incorporation site of impurities into SiC is mainly governed by the covalent radii of these elements as compared to those of C and Si. Roughly speaking, small atoms (light elements) will incorporate on C site while bigger (heavier) ones will do on Si site. It is then intuitive to depict the incorporation behavior by considering a competition between the dopant atom and the matrix atom for a given lattice site. As a consequence, by tuning the C/Si ratio in the gas phase during SiC epitaxy (by chemical vapor deposition (CVD)), one can favor incorporation on one or the other atomic site and thus limit or enhance the incorporation of specific dopants. For example, N incorporation on C site can be significantly reduced (enhanced) by increasing (decreasing) the C/Si ratio in the vapor phase during epitaxial growth. The same rule can be applied to explain the experimentally observed dependence of impurity incorporation on SiC polarity^9^.

Unfortunately, some experimental results cannot be fully explained by the site competition rule. For instance, experimental results suggest that B impurity incorporates on Si site rather than on C site despite a covalent radius of 8.2 pm which is closer to C (7.7 pm) than to Si (11.7 pm) one^10^. In addition, the picture gets more complicated when considering that B was shown to partially incorporate as passivated B-H complex^9^,^11^. More recently, the incorporation of isoelectronic Ge impurity into 4H-SiC does not seem to strictly follow the site competition rule^12^. Indeed, despite incorporating on Si site, [Ge] was found to be independent on C/Si ratio during epitaxy.

To have a full picture of the situation at the growing surface, one has to consider that 4H-SiC homoepitaxial layers can only be achieved through the so-called “step-controlled epitaxy”^13^. This process implies the misorientation of the 4H-SiC (0001) seed of several degrees toward [11–20] or [1–100] directions in order to replicate the polytype of the substrate through a step flow growth mechanism. This is due to the fact that the information on polytype stacking sequence is given at the step edges, not on terraces. As a consequence, since the growth proceeds via step motion, it is commonly considered that the impurities incorporate at the same place as the impinging C and Si atoms, i.e. at the step edges. As a matter of fact, this was so obvious that the experimental studies on the effect of misorientation on impurity incorporation are very few in the literature^14^,^15^,^16^. And the obtained results seem to roughly confirm this idea even though the trends were found to be not very steep and largely dependent on the C/Si ratio and/or on temperature. Note that a recent investigation is showing the contrary, i.e. dopant incorporation is independent on miscut angle and thus on step density^17^.

But this model of impurity incorporation at the step edges does not cover all the experimental observations and the researchers have sometimes difficulties for explaining all the results. For instance, the polarity, i.e. the dandling bonds on the terraces (not at the step edges), is often given as an argument for explaining the observed results^14^,^18^,^19^,^20^. Also, the nature and strength of the chemical bonds formed by the impurities on SiC surface are usually not taken into account for explaining the results^21^.

Starting from the huge accumulation of experimental knowledge on nitrogen incorporation into 4H-SiC, this paper aims at shedding a new light on the incorporation mechanism at the growing surface. We will show that a new model can answer to all the already studied cases and also predict the new ones not studied yet. Extension of the proposed model to other impurities will be addressed in a future paper.

## N incorporation into SiC, an overview

Nitrogen doping is almost exclusively performed using N_2_ gas addition to the deposition chemical system. NH_3_ gas is to be avoided because being too reactive at the growth temperature so that it can lead to SiN_x_ parasitic formation. Also, it is generally assumed that Nitrogen occupies C site mostly because of the similarities in covalent radii between N and C (see Table 1). The hypothesis of N incorporation on Si site for C-face polarity, as proposed by D.J. Larkin^9^, was never really confirmed elsewhere and looks unlikely now. In addition, Si-N bonds are obviously energetically more favorable than C-N ones (see Table 1) so that N should rather incorporate in SiC by making Si-N bonds, that is to say by sitting on C site. This is also supported by the existence of a very stable silicon nitride (Si_3_N_4_) and the absence of any stable C,N-based compound under relevant thermodynamic conditions. As a matter of fact, in this paper, we will consider that N incorporates only on C site.

**Figure 2:**
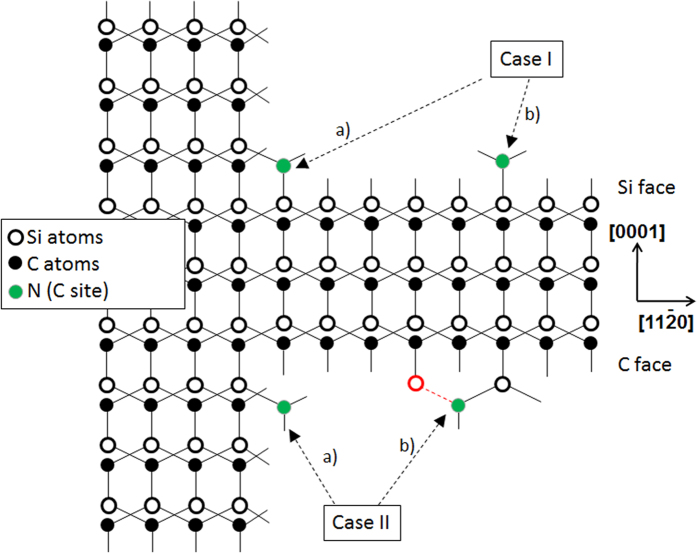
Schematic drawing showing the possible N incorporation sites on both Si and C polar faces of a 4H-SiC crystal.

Let us now list the various possibilities of nitrogen incorporation inside SiC, based on previous works (see for instance refs 14,19), considering both steps and terraces on both C and Si faces. This is summarized in the projection of the crystal structure, represented in Fig. 2. One can differentiate 4 cases:

- On the Si face
  - N incorporates at step edges and forms two bonds with the surface.
  - N incorporates on the terraces and forms only one bond with the surface
- On the C face

- N incorporate at step edges and forms only one bond with the surface.
- N incorporates on the terraces together with one or more Si atoms (in red). N forms one bond with each underlying Si atom.

On the Si face, the less energetically favorable possibility is Ib) case (direct incorporation on terrace) because N creates only one bond with the surface. This mechanism is thus unlikely to occur. Note that it is also inconsistent with the growth by step controlled epitaxy. Indeed, the adatoms participating to the growth are assumed to incorporate at the step edges, not on terraces, or else this should mean the occurrence of 2D nucleation and growth on terraces which should then lead to 3C-SiC inclusion formation.

The other possibility Ia) is the most logical according to the step controlled epitaxy process. N incorporation is favored by the formation of two bonds with the surface. This incorporation mechanism was illustrated by Saitoh *et al*. who showed that the increase of substrate off-angle leads to an increase in N incorporation^15^. Note also that this trend was not very steep and the increase of [N] was in the same order as the increase of misorientation (doubling the misorientation led roughly to 2x[N]). However this trend was found to be C/Si ratio and temperature dependent, i.e. it was effective for C/Si > 1 and T ≤ 1550 °C while the N incorporation was found to be insensitive to off-angle for C/Si ≤ 1 and/or T > 1550 °C.

On the C face, the incorporation at the step edges (case IIa) is not favorable as N creates only one bond. In the case IIb), N is not directly bonded with the surface but via one or more intermediary Si atoms. This should lead again to 2D nucleation and thus favor 3C-SiC polytype inclusions, which is not the case experimentally. As a matter of fact, considering these hypothesis, N should incorporate less on C face than on Si face which is again, experimentally false. The main argument usually proposed for higher N incorporation on C face is the higher number of bonds formed by N with the surface. But one needs to consider a more realistic incorporation mechanism which could allow higher N bonding with the C face without generation of 2D nuclei on the terraces.

This difficulty in proposing an adapted model for N incorporation into 4H-SiC is probably due to the scattering of the reported experimental results, giving rise to somewhat contradictory trends. For instance, some authors reported that N incorporation increases with temperature^15^,^22^ while others mentioned temperature independency^23^ or even a decrease with temperature^14^,^19^. Also, N incorporation on C face (orientation which is known to lead to higher N incorporation levels) is not always found to be strictly higher than on Si face^19^,^23^. Among the arguments used by the authors to explain the discrepancies with other groups’ results, one mainly finds the difference in reactor type (hot or cold wall, vertical or horizontal) and the growth conditions (pressure, temperature, C/Si ratio, growth rate). Though being roughly valid, these arguments are too vague to allow clear identification of the mechanism involved. In order to propose a consistent model, we will first draw a synthesis of the experimental data reported so far by separating the effects of the main four parameters: C/Si ratio, temperature, pressure and growth rate.

### Effect of the C/Si ratio

**Figure 3:**
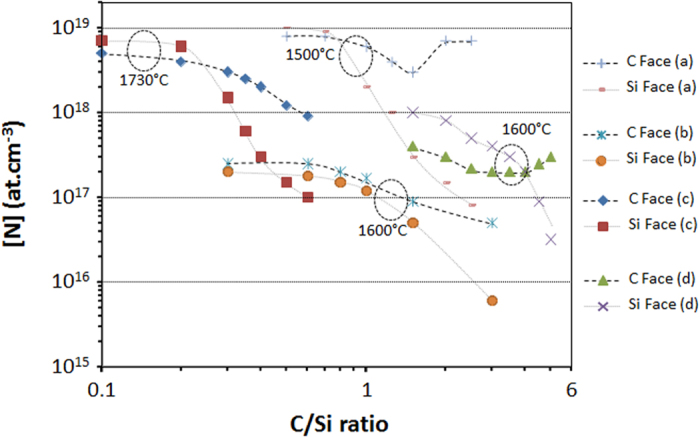
Selected results from the literature showing the N incorporation dependence on C/Si ratio during SiC epitaxial growth. The corresponding references are (a)^16^, (b)^19^, (c)^22^, and (d)^23^.

Most of the works report a decrease of N incorporation when the C/Si ratio increases (see Fig. 3), as expected by the site competition model. As anticipated, C face incorporates more N than Si face but this is only true for the high C/Si ratios. Indeed, at low C/Si ratios (i.e. in Si-rich conditions) N incorporation tends to be very similar for both C and Si faces and even, in most cases, becomes higher on Si face. Authors usually link this surprising result to the specificity of the C-poor growth conditions which generally imply a decrease of the growth rate and/or the formation of Si droplets on the surface. But the mechanism behind these observations is still to be found. Note that C face is always less sensitive to C/Si ratio than Si face. Indeed, the range of variation of N doping level is smaller for the C face.

The boundary between C rich and Si rich conditions (also called “crossover conditions”) is strongly reactor dependent, not only due to the differences of growth conditions (temperature, pressure, growth rate) but also due to differences in hot wall geometry and position of the substrate inside the hot walls.

### Effect of Temperature

**Figure 4:**
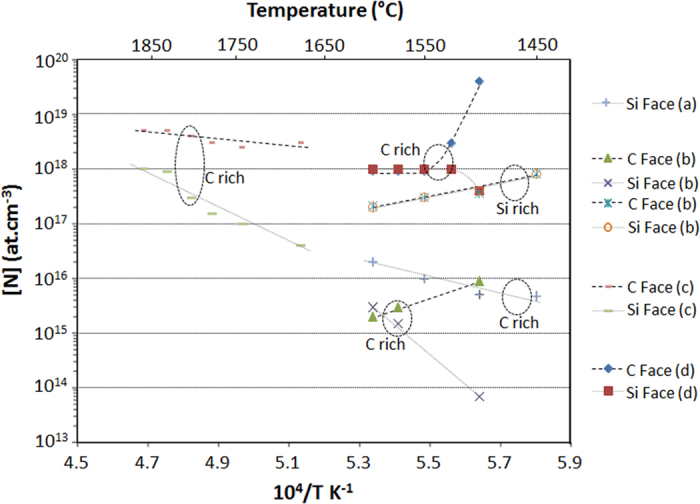
Selected results from the literature showing the N incorporation dependence on temperature during epitaxial growth. The corresponding references are (a)^16^, (b)^19^, (c)^22^, and (d)^23^.

The effect of temperature on N incorporation is less homogeneous from work to work (Fig. 4). For instance, N incorporation can be found either to increase or decrease on both C and Si faces. This might be due to the fact that increasing temperature may modify (decrease) the effective C/Si ratio at the surface due to enhanced C etching by H_2_^16^,^24^. This is probably what is occurring for the results of refs 19,23 shown in Fig. 4 where N incorporation on Si and C faces becomes identical at high temperature which suggests Si-rich conditions (low C/Si ratio) as discussed above. It is thus possible to pass from C-rich conditions at low temperature to Si-rich ones at higher temperature if the nominal (injected) C/Si ratio is not sufficiently high.

Despite this effect, some trends can be extracted from this figure. First, in C-rich conditions (high C/Si ratios) and on Si face, N incorporation is always found to increase with temperature but with varying slopes, and thus different activation energies (E_A_). These E_A_ values are ~60 kcal/mol (calculated from results reported in^16^), 158 kcal/mol^22^ and 260 kcal/mol^19^. All the values of E_A_ being positive, it means that in this particular case, the limiting process for N incorporation is thermally activated so that this incorporation cannot be limited by the desorption of N-containing species or else a negative slope would be found. These values of activation energies are equal or higher than the apparent formation energy one of HCN (~60 kcal/mol^23^) which suggests that, on Si face, the formation of this gaseous species may not be the only limiting parameter to N incorporation. The high activation energy values reported in refs 19,22 are in fact closer to the N_2_ molecule bond strength^25^ while simulation studies are also indicating that the main contributing species to n type doping should be N_2_^26^,^27^. This could be favored by the weakening of the internal N-N bond during adsorption on SiC surface as predicted by theoretical calculations^28^.

But these calculations also predict that N_2_ adsorbs very weakly on the C polar face so that one would expect lower N incorporation on C face than on Si face, which is the opposite to the experimental trends. Also, on C face, E_A_ for N incorporation is not only found smaller than on Si face but also sometimes reported to be negative, i.e. N incorporation decreases with increasing temperature^19^,^23^. N desorption limited incorporation could be this time at the origin of these observations, which is obviously not the case on Si face. Again, the mechanism behind these observations is still to be found.

### Effect of pressure

**Figure 5:**
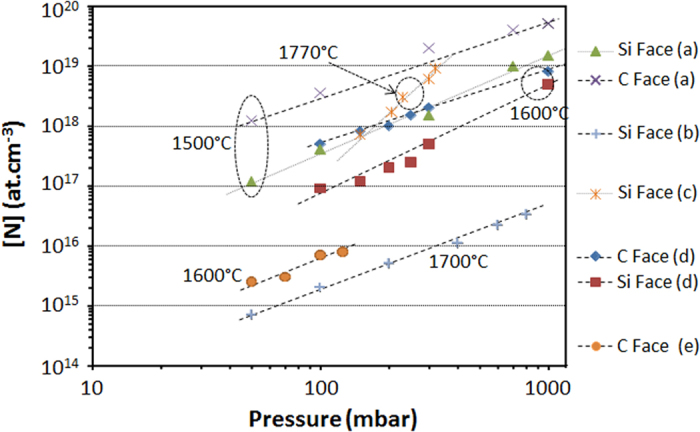
Selected results from the literature showing the N incorporation dependence on pressure during epitaxial growth. The corresponding references are (a)^16^, (b)^17^, (c)^22^, (d)^23^ and (e)^29^.

The effect of growth pressure on N incorporation is at first glance easier to understand since all the curves have the same trend, i.e. [N] increases with pressure increase (Fig. 5). This is ascribed to the decrease of the partial pressure of N-containing species when reducing the total pressure. However, the pressure dependence slopes are not identical from work to work. If one fits each curve according to Henry’s law, i.e. with a power law ([N] ∝ P^1/X^N_X_, where X is the number of nitrogen atoms in the N-containing species responsible for the incorporation), the calculated value of X is varying significantly from 0.29 to 1.1 (see Table 2).

Interestingly, some correlations can be found between these results. For instance, one can see that X values for C face are only slightly fluctuating around 1. But on Si face, X values are systematically found lower than for C face, with a trend toward X reduction when the temperature increases. This suggests that on Si face, the system could be far from a stable state, with a thermally activated process playing a role, this process being absent or less important on C face.

**Figure 6:**
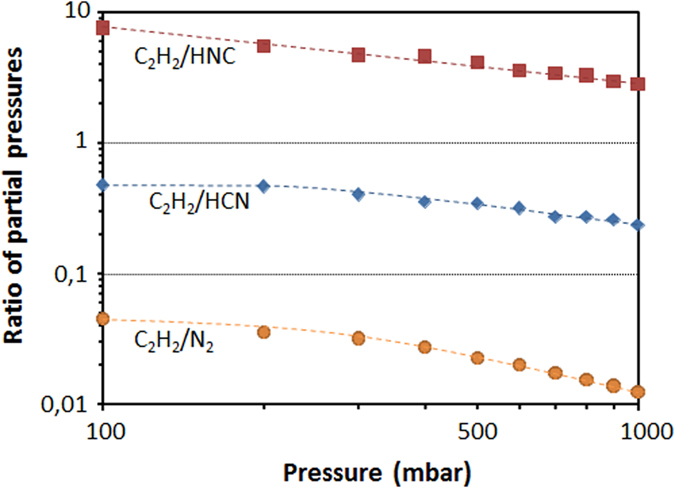
Effect of the growth pressure on the relative amounts of C_2_H_2_ over the main three N-containing species N_2_, HCN and HNC. The curves are extrapolated from the data of^23^.

It was suggested from the temperature dependence results that N_2_ could be the main species responsible for N incorporation into SiC and thus X value in the power law should be close to 2. This is not the case experimentally. Other parameter should be thus also considered. Zhang *et al*.^22^ proposed that the growth rate, which usually increases when pressure decreases^29^, can further influence N incorporation, as will be discussed later. Forsberg *et al*.^23^ attributed this discrepancy with Henry’s law to a change of the carbon coverage over the growing surface. This was supported by thermodynamic calculations showing that the ratio between C_2_H_2_ (the commonly assumed active C-containing species) and HCN or HNC partial pressures is increasing when decreasing pressure (see Fig. 6). In this figure, N_2_ molecule is also considered but it does not change the general trend. So, according to thermodynamic calculations, decreasing growth pressure favors C enrichment of the gas phase relatively to N (increase of the C/N ratio), for all the N-containing species envisaged, so that N incorporation into SiC should decrease according to site competition rule. Note that this calculated increase of C/N ratio at low pressure cannot be directly translated in terms of increase of C/Si ratio though it goes in the same direction. By extension, C face being less sensitive to C/Si ratio as seen earlier, this increase in C coverage at lower pressure should be less effective on C face than on Si face. This would explain the higher values of X and the lower sensitivity of X toward temperature for C face (Table 2).

One can also consider some simple physico-chemical effects influencing the effective C/Si ratio at the surface. Indeed, reducing the pressure leads automatically to reduced H_2_ partial pressure, and thus to less removal/etching of C atoms from the SiC surface. At the same time, Si evaporation from the surface is enhanced at reduced pressure so that both effects go in the same direction toward the increase of effective C/Si ratio on the surface at lower pressure. This could also contribute to the reduced N incorporation at lower pressure due to local increase of the C/Si ratio.

As a matter of fact, the effect of growth pressure on N incorporation is not as simple as it seems since this parameter is also affecting significantly both the growth rate and/or the C/N ratio at the surface.

### Effect of growth rate

**Figure 7:**
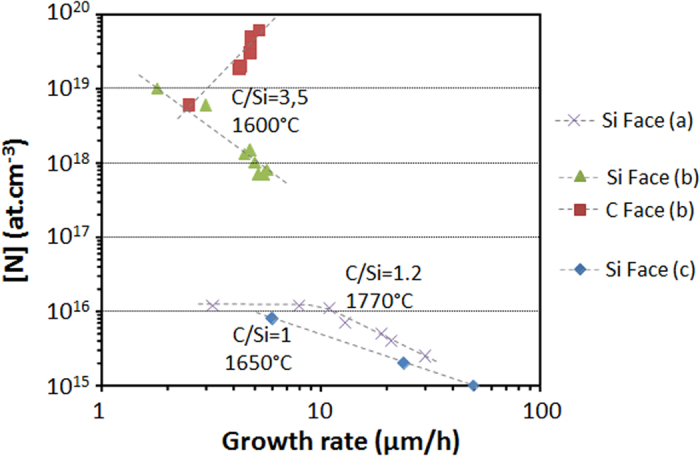
Selected results from the literature showing the N incorporation dependence on growth rate during epitaxial growth. The corresponding references are (a)^17^, (b)^23^ and (c)^31^.

The effect of growth rate on N incorporation has been less documented (see Fig. 7). C face seems to behave in an opposite way to Si face, i.e. increasing growth rate decreases [N] on Si face while it increases [N] on C face^23^. In order to better understand the processes involved, one should consider the simulation results from refs 24,30, where it is shown that the local C/Si ratio on the seed surface does not keep the original injected value and is silane flux, and thus growth rate dependent. For injected C/Si >1, calculations shows that the C/Si ratio at the surface increases quasi-linearly with growth rate despite the fact that the inlet C/Si is kept constant. This local increase in C/Si ratio at higher growth rate can explain the decrease in N incorporation on Si face since this polarity is very C/Si dependent as shown in Fig. 3. C face may act differently because, on this polarity, N incorporation was found to be less sensitive to C/Si ratio. But this argument does not look sufficient to explain the opposite trends observed between each face.

## A new model of nitrogen incorporation

Having reviewed and partially discussed the main experimental trends found for N doping of 4H-SiC during epitaxy, the most striking is the predominant effect of the C/Si ratio. Indeed, for at least two other parameters (temperature and growth rates) some of the observed trends can be explained by a correlated modification of the local C/Si at the surface. But with the currently accepted model (schematized in Fig. 2), the effect of the C/Si ratio is by far the less explained. One needs thus to build up a model which puts the local C/Si ratio at the surface at the center of the process leading to N incorporation into 4H-SiC. To get a global description of the surface composition as a function of process parameters, it is important to consider elementary steps, not only condensation (related to the incoming fluxes of species) but also vaporization from the surface. This latter phenomenon occurring when heating up a material sufficiently high, is often underestimated. In the case of SiC, which exhibits a non-congruent vaporization, it is well-known that when heating up under inert ambient (either Ar or vacuum) the flux of Si atoms (i.e. Si-containing species) emitted by the surface is higher than the one of carbon which leads to C enrichment (graphitization) of the surface. This process has been advantageously used for the growth of epitaxial graphene^32^,^33^. But if SiC is heated under a H_2_ ambient, then the vaporization rate of C from the surface (via the formation of hydrocarbons) turns to be higher than the one of Si, leaving Si droplets on the SiC surface^34^.

When an atom vaporizes from a surface, it leaves behind a vacancy in the solid material at the solid/gas interface. But if the solid is in contact with a vapor phase already containing the element to be vaporized (under the form of gaseous species for instance), then the elemental loss can be partially or completely compensated by the gaseous feeding, leaving an apparently stabilized surface. Differently said, depending on the nature and the composition of the vapor phase applied to the crystal surface, the vaporization/condensation reactions ratio of each element can be shifted or even balanced. This is the case when heating up SiC under a mixture of H_2_ and hydrocarbon. Indeed, it is known that the presence of hydrocarbon avoids Si droplets formation at high temperature and reduces significantly SiC etching rate^34^,^35^,^36^. In fact, this carbon over-pressure on the SiC surface is, by nature, a dynamic process, in the sense that C atoms (or C-containing species) constantly collide with the crystal surface. It should thus constantly exchanges C atoms with the surface: the cracking of hydrocarbons brings elemental C to the surface which are compensating for the C atoms etched away by H_2_ from SiC surface. As a matter of fact, introducing in the gas phase an impurity which can replace carbon inside SiC matrix, N for instance, can lead to some N incorporation by this constant surface exchange of atoms between the solid surface and the gas phase. Differently speaking, N incorporates when a C site is free of C at the surface, that is to say when a C vacancy (formed by H_2_ etching) is available at the surface. This vacancy driven N incorporation is the main idea of the proposed model which is meant to take into account not only incorporation at the step edges but also on the terraces. Actually, when considering a non-congruent vaporization, the atomic fluxes are not emitted only at the step edges but also on the terraces (for instance when graphene forms, it covers the entire SiC surface or when Si droplets form, they do not only occur at the step edges). The same comment can be done for the reverse reaction as the whole crystal surface is collided by gaseous species. Note that such exchange mechanism has never been considered yet for explaining the experimental trends observed for impurity incorporation into SiC during epitaxy. Also, it is important to mention that this kind of incorporation does not participate strictly speaking to the growth itself because the incorporating atoms are just replacing some losses.

**Figure 8:**
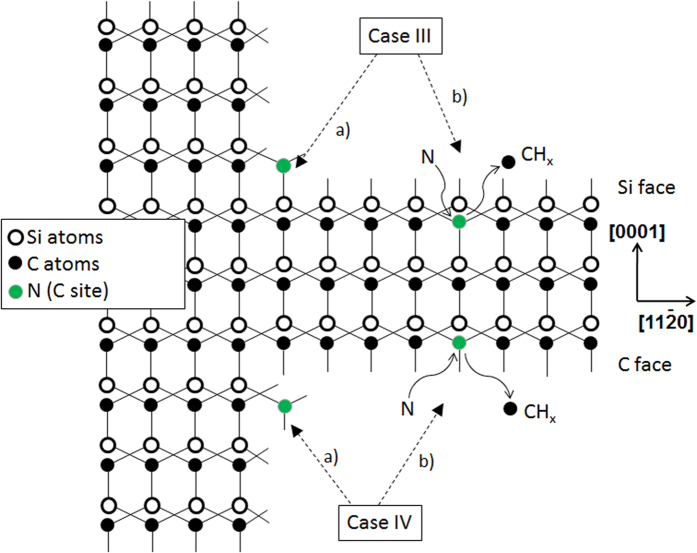
Schematic drawing summarizing the proposed model for N incorporation on both Si and C polar faces of a 4H-SiC crystal.

This model is summarized in Fig. 8 which is in fact an actualization of Fig. 2 taking into account N incorporation via a C vacancy. For N incorporation on the terraces, attachment of N and/or Si atoms on top of the terraces (cases Ib et IIb in Fig. 2) which were considered unlikely has been replaced by direct N insertion inside the SiC material via a surface (case IVb) or sub-surface (case IIIb) C vacancy. Note that the latter case IIIb, where a C vacancy forms below the top atomic surface, is not ruled out in order to keep this hypothesis for the discussion. But when making the parallel with conditions of graphene growth on SiC by preferential Si loss, which occurs in similar temperature range and which implies also vacancy formation below the surface, the present assumption assuming some C loss below the surface does not appear so improbable if the C overpressure is not sufficient.

Considering now these “new” sites of N incorporation, one can see that in both cases IIIb and IVb N is directly linked to SiC via four and three bonds respectively. These sites of incorporation are thus obviously much more stable than just bonding on top of a SiC surface.

Finally, since the growth is exclusively occurring at the step edges (step-controlled homoepitaxy conditions), the exchange balance at the steps between the gas and the surface should be locally shifted toward atoms incorporation instead of atoms loss (in other words, the mean time for H_2_ etching and thus C vacancy formation is much reduced on step edges). We will thus neglect the possibility of C vacancy-induced N incorporation at the step edges and will consider only the standard incorporation process in these places.

## Discussion

The present model proposed two new sites of N incorporation, depending on SiC polarity. Obviously, the case IVb (on C face) needs less energy to occur because the C atom leaving the surface is directly in contact with the gas phase and it is bonded to the crystal with three bonds instead of four in the IIIb case (on Si face). As a matter of fact, this simple energetic consideration allows explaining why C face incorporates more N than Si face does (in C-rich gaseous conditions). But when the C/Si ratio becomes too low, the experimental results show that both polar faces incorporate almost identical amount of N, that is to say that the C vacancies on both faces have rather similar occurrence probability. Once adsorbed in a carbon vacancy, the N atom shows a lower desorption probability on the Si-face than on the C-face because the incorporation sites are “buried” under the surface. This could explain why at low C/Si ratio, [N] can be even higher on the Si-face than on the C-face (see Fig. 3). Low C/Si ratios are very specific conditions in which the C overpressure above the SiC surface is too low so that the equilibrium of atomic exchange between the surface and the gas phase could be shifted toward partial C loss from the solid. As a matter of fact, in these Si-rich conditions, the growth rates are generally reduced which, according to Fig. 7, goes toward the increase of N incorporation in the grown crystal.

Coming now to the effect of temperature, the experimental results suggest that N incorporation on Si face is not limited by N-containing species desorption from the surface since E_A_ is always found positive. In other words, a thermally activated process is playing a major role on Si face while being almost absent on C face. According to our model, the same energetic explanation as for the C/Si ratio can be used: forming a C vacancy on Si face is obviously energetically more difficult than on C face. As a matter of fact, apparent activation energies for N incorporation would be lower on C face than on Si face because of the difference of formation energy of these vacancies between each face.

As mentioned earlier, theoretical calculations were predicting that N_2_ molecule adsorbs very weakly on the C face^28^ which direct conclusion would lead to opposite trend (lower N incorporation on C face than on Si face) compared to the experimental ones. But these calculations were only considering a “perfect” SiC crystal surface, i.e. without the possible presence of C vacancies. These C vacancies on C face would increase the sticking coefficient of N_2_ molecules by proposing incorporation sites with 3 bonds to the matrix which could further catalyze N_2_ adsorption and/or cracking. As a consequence, if adsorption or cracking is playing a role, then increasing temperature should affect N incorporation rate. Since [N] in SiC mostly decreases with increasing temperature on C face, then N_2_ desorption should be the limiting process on this polar face and not N_2_ cracking.

To summarize the effect of temperature, N incorporation on C face is influenced simultaneously by the availability of C vacancies on the terraces and the kinetic of N desorption from the surface while on Si face the availability of C vacancies is the main limiting factor. This difference on C vacancy availability between the two polar faces may also contribute to the differences observed as a function of growth pressure, for instance the decrease of X parameter with increasing temperature on Si face (see Table 2). Also, the fact that N desorption from the surface is a limiting factor only for C face explains why N incorporation increases with increasing growth rate. Indeed, increasing the growth kinetics should reduce the time allowed for N desorption and thus favor N incorporation. On Si face, the absence of such N desorption limitation and the high sensitivity to C/Si ratio results in the reverse trends with increasing growth rate.

The proposed model considers that terraces are contributing more to N incorporation than step edges without excluding it. Most probably, both C vacancy assisted N incorporation on terraces and direct incorporation at step edges are occurring at the same time. In order to have a full picture of the situation, one should also consider the geometric aspects of the 4H-SiC(0001) growth surface. Indeed, such surface is ideally composed of an array of parallel steps and terraces whose dimensions depend mainly on the substrate off orientation. For instance, assuming an average 1 nm step height (one 4H-SiC unit cell along the c axis), the average terrace width can be deduced using simple geometric calculation : it is ~14 nm and ~7 nm for 4° and 8° off-orientation respectively. It means that, in the case of an ideally uniform step and terrace structure, the solid/gas interface is composed of ~94% of terraces and of only ~6% of step edges on top of 4° off seeds. This proportion only slightly evolves to 87.5% terraces/12.5% step edges for 8°off seeds. As a matter of fact, even if steps and terraces are both participating to N incorporation, the processes involved on terraces would play a prominent part. As an illustration, according to ref. 15, increasing step density leads to N incorporation increase (by direct incorporation at the step edges). But as said earlier, this effect is rather moderate compared to the effect of the growth parameters discussed in this paper and which effects can be better explained by processes occurring on terraces.

The main hypothesis of the present model is that some C vacancies can form at the surface during epitaxial growth. Though *in situ* direct observation or detection of these surface vacancies would be very difficult, C vacancies in the 4H-SiC crystals are usually detected after epitaxial growth, using mainly Deep level Transient Spectroscopy (DLTS) technique^37^,^38^. These point defects were associated to the well-known Z_1/2_ centers^39^ which density was found to increase when the C/Si ratio decreases^40^ or the temperature increases^41^ during the growth on Si-face seed. Interestingly, these trends for Z_1/2_ centers formation are similar with the conditions of high N incorporation on Si face. But one should also consider that the concentration of Z_1/2_ centers was always found lower than ~10^15^ cm^−3^, without direct correlation with the level of N incorporated. It means that, even if C vacancies at the surface can allow massive N incorporation inside the SiC matrix (up to > 1 × 10^19^ at.cm^−3^), their formation is just a transient surface problem. This is consistent with the present model involving constant exchange of C atoms between the solid and the gas phases, i.e. involving transient C vacancy formation by H_2_ etching followed by filling of these vacancies by C (or N) atoms from the gas phase. Note that the value reported for the formation energy of C vacancies in SiC, ~5 eV/atom (~115 kcal/mol)^42^,^43^, is in the range of activation energies reported for N incorporation evolution with temperature (60 to 260 kcal/mol). But care should be taken when comparing directly these values since they most probably do not relate to the same mechanisms of C vacancy formation: surface versus bulk, H_2_ etching versus self-generation.

Even if the results discussed in this paper are only coming from epitaxial growth, a supporting argument comes from sublimation growth. Indeed, the large area commercial wafers display very frequently a darker central zone ascribed to local higher N incorporation level. These areas of higher N incorporation are usually correlated to the (000 ± 1) natural facets of the boules during bulk growth by the Physical Vapor Transport (PVT) method. In other words, areas displaying almost on-axis orientation, and thus with large terraces, are subjected to higher N incorporation. This doping difference can be explained with our model by the increased availability of C vacancies at the surface on large terraces (In this case C atoms are vaporized not though CH_x_ species formation but only by the very high temperature used in the PVT process). This point will be investigated more systematically in a future paper.

Finally, though the ambitious goal of the present model is to try proposing a universal explanation of all the experimental results gathered so far on N incorporation inside 4H-SiC during CVD growth, it cannot address an important experimental factor: reactor dependency. For sure, each reactor has his own working conditions (and calibration curves) and one cannot just transpose these conditions from one reactor to the other, even if of the same type and/or geometry. This reactor dependency has increased the scattering of the results (doping ranges, slopes, activation energies…) and rendered more difficult the finding of a proper general model for all of them. We believe that the surface vacancy-induced N incorporation proposed in the present paper gives a reasonable general frame for all the reported data so far, even beyond the reactor dependency. We even can speculate that the physical mechanisms for vaporization/condensation at the SiC surface being similar under the same set of physical parameters, the deviations between the different author’s results (as reported in Figs 3 to 6) constitute an indirect “characterization” of the reactors themselves and how they affect the real fluxes of species coming to and leaving the surface.

## Conclusion

The proposed surface exchange model allows explaining the main trends experimentally observed for N incorporation into 4H-SiC during homoepitaxial growth, even the apparently contradictory ones. It implies the transient formation of C vacancies at the surface (on C face) or at the sub-surface (on Si face) which are statistically formed during the dynamic exchange occurring at the gas/solid interface at the temperatures of growth under H_2_ ambient. Discussing the model, we also demonstrated that C/Si is by far the dominant parameter for N incorporation as it directly impact the carbon vacancy formation. All the other parameters (temperature, pressure and growth rates) are mainly modifying the local C/Si at the surface. This model can virtually be used for all types of reactors and to all impurities incorporating on C atomic site. In that sense, it is more global than the “site competition mechanism” because it addresses the nature of the incorporation sites, and how those sites can evolve with the physical parameters of the process.

## Additional Information

**How to cite this article:** Ferro, G. and Chaussende, D. A new model for *in situ* nitrogen incorporation into 4H-SiC during epitaxy. *Sci. Rep.* **7**, 43069; doi: 10.1038/srep43069 (2017).

**Publisher's note:** Springer Nature remains neutral with regard to jurisdictional claims in published maps and institutional affiliations.

## Figures and Tables

**Figure 1:**
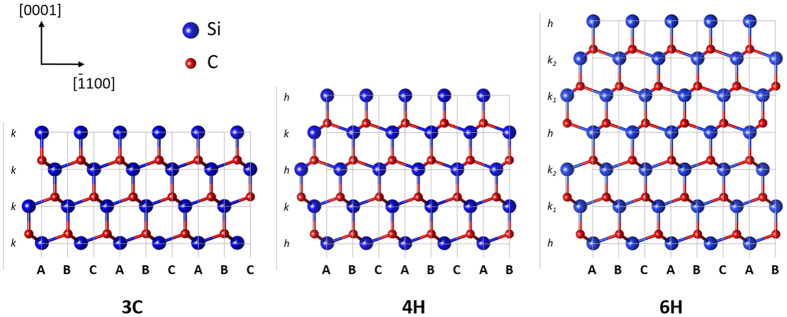
Illustration of SiC polytypism; Si-C bilayer stacking along the c [0001] axis for the three main SiC polytypes 3C, 4H and 6H. h and k stand for hexagonal and cubic type of stacking respectively.

**Table 1:** Thermodynamic data considered in the present work on the Si-C-N ternary system.

	Covalent radius (nm)	Bond in SiC	Stable compound	Melting/Sublimation point (°C)^44^^ ^*	Gibbs free energy of formation (kJ/mol) at 300 K	Enthalpy of formation (kJ/mol) at 300 K
C Si	0.077 0.110	Si-C	SiC_(s)_	2100 (S)		−73^45^
N	0.075	N-Si	Si_3_N_4(s)_	1900 (S)		−744^45^
		N-C (H)C-N	CN_(g)_ HCN_(g)_	—	+404^45^ + 124^45^	+435^45^ + 135^45^

^*^(S) = sublimation

**Table 2:** Evolution of the fitting parameter X of the curves in Fig. 5, using a power law ([N] ∝ P^1/X^N_x_), as a function of other conditions.

Polarity	Temperature (°C)	X	Ref
C face	1500	0.83	16
C face	1600	1.1	23
C face	1600	0.72	30
Si face	1500	0.67	16
Si face	1600	0.5	23
Si face	1700	0.68	17
Si face	1770	0.29	22
